# Sustainable Natural Deep Eutectic Solvent-Mediated Synthesis of Magnesium Zirconate Nanoparticles: A Photocatalyst for the Degradation of Anti-Viral Drug

**DOI:** 10.1021/acs.inorgchem.4c03383

**Published:** 2024-10-11

**Authors:** Balasubramanian Sriram, Abhikha Sherlin V, Sea-Fue Wang, Jackulinflora P, Mary George

**Affiliations:** † Department of Materials and Mineral Resources Engineering, National Taipei University of Technology, No. 1, Section 3, Chung-Hsiao East Road, Taipei 106, Taiwan; ‡ Department of Chemistry, Stella Maris College, Affiliated to the University of Madras, Chennai, Tamil Nadu 600086, India

## Abstract

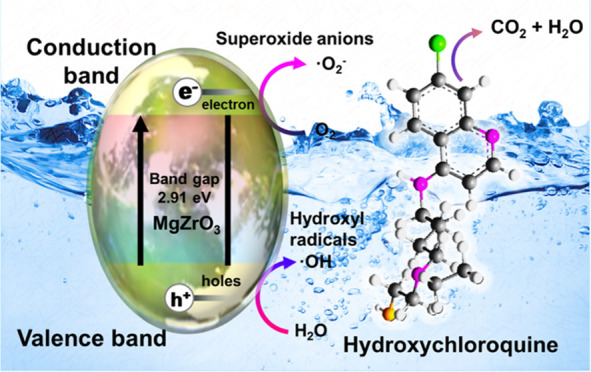

The anti-viral drug hydroxychloroquine (HCQ) has captivated significant interest in the pharmaceutical field, as it is a quinolone derivative. Its unrestrained occurrence causes prominent health hazards owing to its persistent, carcinogenic, recalcitrant, and teratogenic nature. Herein, in this work, an experimental investigation was carried out toward the photocatalytic degradation of HCQ drug using magnesium zirconate (MgZrO_3_) nanoparticles as an effective photocatalyst. A comprehensive characterizations of the as-synthesized material was carried out. The photocatalytic degradation of the HCQ drug was examined with various sources of light energies. The obtained outcomes indicated that ±85% of HCQ was degraded using a MgZrO_3_ photocatalyst within 30 min of the reaction time under UV–visible (ultraviolet) light irradiation. Further, other significant operational parameters such as various catalyst dosages, HCQ concentrations, pH, scavengers, and salts were examined. The degradation studies revealed that the reaction followed pseudo-first-order kinetics. Hence, this perovskite-type MgZrO_3_ has grasped profound attention in environmental remediation, significantly in photocatalytic degradation of HCQ drug. This comprehensive research offers green synthesis strategy as a substantial framework for providing effective photocatalyst that addresses contemporary water pollution issues linked to notable results. This aids in targeting era-driven advancements toward a clean and safe future environment.

## Abstract 2

Herein, in this work, an experimental investigation was carried out toward the photocatalytic degradation of hydroxychloroquine using magnesium zirconate nanoparticles as an effective photocatalyst.

## Introduction

The prompt growth of the drug industry has resulted in the discharge of pharmaceutical contaminants into various water systems, making the water unfit for human and animal consumption. The utmost challenge faced by the global market is water pollution.^1^ The persistence of drugs and their harmful metabolites in aquatic matrices has been regarded as a major environmental concern. Further, owing to its contribution toward global warming and its negative effects on human health, it increases fatality rates worldwide.^2−5^ Pharmaceutical metabolites that enter water systems alarm the ecosystem.^6,7^ Particularly, hospital, industry, and landfill effluent may include large amounts of pharmaceuticals that contaminate rivers, lakes, groundwater, and drinking water.^8−11^ The antimalarial drug hydroxychloroquine (HCQ) stands as a significant drug owing to its potential use in COVID-19 treatment.^12−14^ The US Food and Drug Administration authorized an emergency use authorization for HCQ toward nonclinical trial COVID-19 patients on March 30, 2020.^15^ This has increased its usage worldwide, which has been further exacerbated by the unadvisable habit of self-medication. Long-term use of HCQ drugs has a serious influence on the retina and cornea of the eyes.^12^ The capability of the HCQ drug to alter the pH at the cell membrane surface prevents the virus from adhering to the membrane.^16^ The detrimental impact of this HCQ drug on the environment includes the emergence of antibiotic-resistant bacteria and cardiotoxic effects. Therefore, it is important to abate the target drug species from water using the appropriate analytical methods. da Silva et al. reported the potential of natural zeolite clinoptilolite-supported zinc oxide catalyst toward the photocatalytic degradation of the HCQ drug. Catalysts were prepared using the wet impregnation technique, and the maximum percentage of degradation was reported.^17^ Similarly, Priya et al. have reported the synthesis of tungsten trioxide nanorods/nitrogen-doped carbon nanofiber nanocomposite and its usage in electrochemical sensing HCQ. The study examined the excellent reliability of the nanocomposite for sensing HCQ in real-time samples such as wastewater and urine.^18^

Conventional treatments to purify water cannot entirely eradicate the HCQ drug pollutant, thereby complicating our efforts to meet the sustainable development target for clean water. Recently, the cutting-edge photocatalytic degradation approach has grown in popularity because of its low cost, ease of disposal, energy efficiency, and environmental friendliness.^19,20^ Especially, the photocatalytic degradation technique uses a potential photocatalyst to mineralize the target drug species to CO_2_ and H_2_O molecules. Further, this technique holds several merits including enhanced degradation rate, simple procedures, no formation of harmful byproducts, profitable commercial use, and negligible disposability. An efficient photocatalyst must participate in the principal deactivation process of electron–hole recombination.^21−23^

Nanomaterials have drawn significant attention over the past decade. Their use in photocatalysts, electronic devices, drug delivery, biosensors, and microwave devices is due to their remarkable characteristic features.^24^ Perovskite materials have emerged as the most appealing and cost-efficient energy materials for various photocatalytic applications.^25^ Perovskites are fascinating because of their physicochemical characteristics, such as electron mobility, redox behavior, and thermal stability, making them ideal candidates for various applications in water-splitting catalysis, optical devices, and solar cells.^26^ Significantly, metal oxide nanomaterials demonstrated remarkable photocatalytic efficacy for the elimination of contaminants, attributed to their increased surface energy, elevated surface area, quantum confinement effects, and remarkable physical and chemical features, in contrast to conventional bulk materials. Apart from these properties, these metal oxides are found in abundance and exhibit excellent stability in a wide range of conditions. Nonetheless, a noteworthy drawback of employing metal oxide photocatalysts is the rapid recombination of the photogenerated electron–hole pairs, which results in an inept production of free radicals necessary for the photocatalytic degradation of drugs.^27^ This issue can be addressed appropriately by adjusting the band gap of the metal oxides. In this regard, zirconia-based nanoparticles are considered an exceptional material of choice for photocatalytic applications owing to their porosity, chemical inertness, increased surface area, thermal stability, tunable band gap, and cost-effectiveness. However, zirconia nanoparticles suffer from a fast recombination effect. To combat this issue, magnesium ions attract attention among researchers as the oxide form of magnesium benefits from enhanced defect centers that generate O_2_^–^ ions. These O_2_^–^ ions upsurge the photocatalytic activity of zirconia.^28^ In this regard, MgZrO_3_ is considered an efficient material for photocatalytic applications. Magnesium zirconate (MgZrO_3_) is an ABO_3_-type perovskite material with excellent electrical and optical properties. MgZrO_3_ has more trap centers (oxygen vacancies), which reduces the band gap; thus, it is considered a potential catalyst for carrying out photocatalytic reactions.^29^ MgZrO_3_ requires relatively inexpensive, environmentally benign, and simple preparation processes.

Deep eutectic solvents (DESs) are being investigated for excellent potential in material synthesis as they are cheap, environmentally friendly, and biocompatible. Further, a particular class of DES solvents known as natural deep eutectic solvents (NADES) are recognized as alternatives to room temperature ionic liquids, supercritical fluids, and subcritical fluids, respectively. In view of environmental perspectives, thymol–menthol NADES are terpenes that result in renewable solvents.^30^ Significantly, the choice of thymol–menthol NADES in the synthesis of MgZrO_3_ offers an enhanced hierarchical architecture associated with excellent characteristics. The complete energy requirement for the synthetic procedure is brought down drastically using this solvent.^31^ Recently, thymol–menthol NADES has gained huge popularity, for their usage as a solvent medium in the synthesis of various perovskite materials.^32^ Eventually, this will result in the advancement of the photocatalyst structure and properties that are capable of degrading the HCQ drug.

**Scheme 1:**
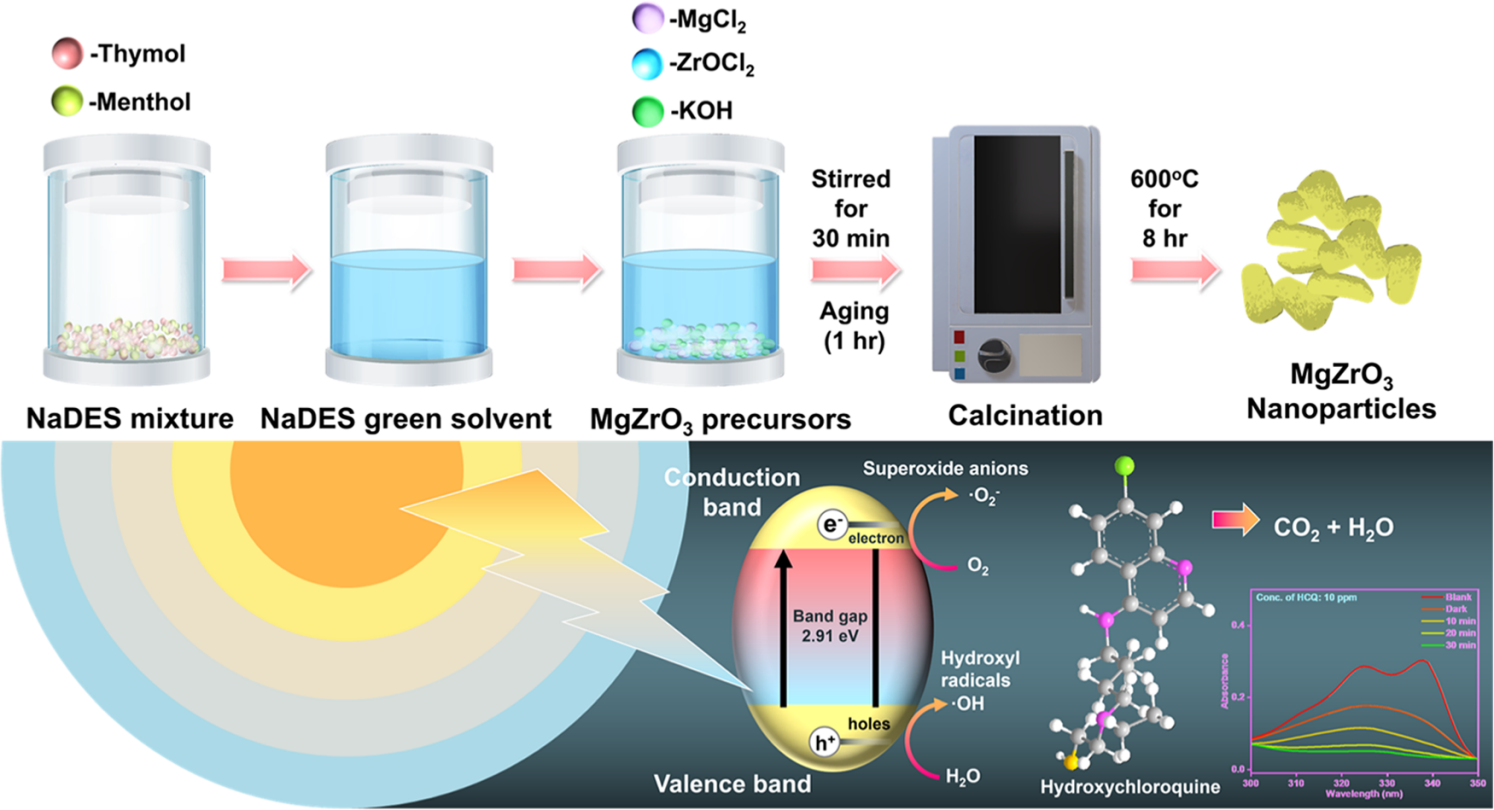
Schematic Illustration of Green Solvent-Based NADES-Assisted Synthesis of MgZrO_3_ Catalyst for Photocatalytic Degradation of HCQ

Therefore, we propose a simple and cost-effective preparation route for the synthesis of perovskite-type MgZrO_3_ nanomaterial using the thymol–menthol NADES-mediated coprecipitation method as illustrated in Scheme 1. The use of a thymol–menthol sustainable solvent in the synthetic procedure offers a well-defined MgZrO_3_ structure that discloses the active surface sites for outstanding photocatalytic performance. The proposed photocatalyst is characterized meticulously for structural, functional, and morphological analysis. Furthermore, the as-made MgZrO_3_ was employed as an efficient photocatalyst for HCQ degradation studies. The HCQ degradation efficacy of alkali metal-based zirconate was analyzed under various experimental conditions, such as the effect of catalyst dosage, the concentration of HCQ solutions, scavengers, salts, pH, and light sources. To the best of the authors’ knowledge, no previous use of NADES-mediated MgZrO_3_ nanoparticles has been reported as a potential ideal candidate for the degradation studies of HCQ drugs.

## Materials and Methods

### Chemicals and Reagents

Thymol (C_10_H_14_O), menthol (C_10_H_20_O), and zirconium oxychloride octahydrate (ZrOCl_2_·8H_2_O) were purchased from Loba Chemie Pvt., Ltd. Magnesium chloride hexahydrate (MgCl_2_·6H_2_O) was bought from Fisher Scientific Pvt. Ltd. Methanol (CH_3_OH) and potassium hydroxide (KOH) were purchased from Avantor. All of the chemicals purchased were of analytical grade and used as received. Hydroxychloroquine (HCQ, 200 mg) tablets were purchased from Apollo pharmacy. All of the solutions needed for the photocatalytic degradation studies were made up of distilled water. Glassware was washed with aqua regia and ethanol, rinsed with deionized water, and dried before use

### Preparation of NADES

Typically, 4.5 g of thymol and 4.7 g of menthol were placed in a glass beaker with a magnetic pellet. The mixture was combined by stirring continuously until a homogeneous transparent solution was obtained at room temperature.

### Preparation of MgZrO_3_

To synthesize MgZrO_3_ nanoparticles, 0.05 M (0.407 g) of MgCl_2_·6H_2_O and 0.05 M (0.644 g) of ZrOCl_2_·8H_2_O were dissolved in 10 mL of CH_3_OH, respectively, added to 10 mL of thymol–menthol NADES, and stirred at room temperature. This was followed by the dropwise addition of 6 M KOH (precipitation agent) to the above solution. The solution was subjected to continuous stirring, which resulted in the formation of MgZr(OH)_6_ hydroxides. The resultant mixture was aged for 1 h. Further to eliminate undesirable ions, the aged solution was rinsed with double-distilled water thrice, filtered, and dried in a hot air oven at 80 °C overnight. The resultant white precipitate was placed in a crucible and calcinated for 8 h at 600 °C. The schematic image representing the preparation method is given in Scheme 1.

### Photocatalysis of HCQ

The photocatalytic degradation application was examined using 10 ppm HCQ (100 mL) solution. The as-made photocatalyst MgZrO_3_ (100 mg) was added to 100 mL of HCQ solutions. The solution was magnetically stirred in the dark for 30 min. During this period, the solution attains an adsorption–desorption equilibrium. After 30 min, the photocatalytic degradation experiment of the HCQ drug molecule was conducted using a UV–visible light source. The liquid drug suspensions (4 mL) were withdrawn from the photocatalytic reactor every 10 min, respectively. The absorption spectra of the degraded HCQ solution were recorded using a UV spectrophotometer.

### Characterization Studies

To investigate the purity and the crystal phases present in the as-made samples, powdered X-ray diffraction (PXRD) technique was employed. The diffraction patterns were recorded using an X-ray diffractometer (Bruker AXS D8 instrument) with Cu Kα radiations. PerkinElmer model spectrum RXI was utilized to record the infrared spectrum from 400–4000 cm^–1^ using a KBr pellet. The textural surface characteristics were examined by using a scanning electron microscope (FEI Quanta FEG 200). The diffuse reflectance UV spectrum for the as-made sample was recorded using a Deepvision 2373 UV spectrophotometer from wavelengths of 200–800 nm at room temperature.

## Results and Discussion

### Characterization of the As-Synthesized Perovskite Material

#### X-ray Diffraction (XRD) Analysis

**Figure 1:**
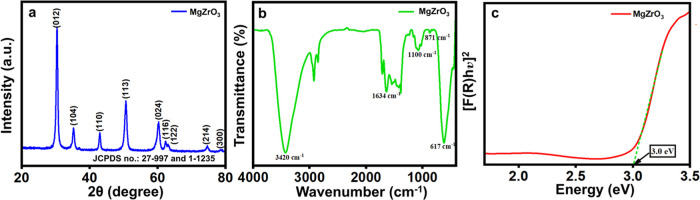
(a) XRD patterns, (b) FTIR spectrum, and (c) UV-DRS Tauc plot of MgZrO_3_ nanorods.

The XRD diffraction pattern of the as-synthesized magnesium zirconate powder is given in Figure 1a. The XRD pattern revealed the formation of magnesium zirconate crystal planes. The patterns were predominantly zirconia (cubic phase), with a minor influence from cubic magnesium oxide. The structure was strikingly identical to the equivalent standard JCPDS card nos. 27–997 and 1–1235. The absence of additional impurity peaks in the XRD data showed that the as-prepared material has a high purity. XRD patterns revealed that several reflection peaks concerning the relevant crystalline planes of (012), (104), (110), (113), (024), (116), (122), (214), and (300) were observed in the XRD spectra at the different diffraction angles of ≈30.3, 35.2, 42.9, 50.6, 60.2, 62.3, 63.0, 74.6, and 78.5°, respectively. The inclusion of the thymol–menthol solvent is the main cause for the high purity of the sample that formed at low calcination temperature. The traces of other impurities and phases of oxides are found to be absent. This is accredited to the synthetic procedure followed for the preparation of MgZrO_3_ nanomaterials. Further, the Debye–Scherrer formula was used to calculate the average crystalline size (eq 1):
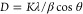
1where *D* denotes the size of the particle diameter, *K* denotes the Scherrer constant usually calculated as 0.9, λ represents the X-ray source wavelength, β denotes the full width at half-maximum (fwhm), and θ represents the diffraction angle of the lattice plane. The average crystallite size was found to be ≈65.23 nm.

#### Fourier-Transform Infrared Spectroscopy (FT-IR) Analysis

The FT-IR spectral studies record the molecular interactions and the absorption vibrations of the as-synthesized MgZrO_3_ nanoparticles to confirm the functionalities present on the surface of the material. The FT-IR spectrum of as-made MgZrO_3_ is displayed in Figure 1b. The vibrational modes associated with the metal–oxygen bonds such as Mg–O and Zr–O are indexed to the broad peak present in the ≈460–775 cm^–1^ region. Mg and Zr in their hydroxide state are accountable for the wide peak at around ≈800–1000 cm^–1^. Asymmetric Mg–O–Zr stretching is confirmed by the peak stationed at ≈1100 cm^–1^. A significant broad peak at ≈3420 cm^–1^ is related to the stretching vibrations of environmental absorbed −OH involving Mg–OH and Zr–OH, whereas the peak at ≈1634 cm^–1^ is linked to the bending vibration of water molecules. The peaks at ≈1370–1350 and 3000–2850 cm^–1^ are due to the C–H stretching obtained from methanol. The inclusion of the green solvent causes exact functionalization of the MgZrO_3_ nanomaterial and excludes the presence of other functional groups that disturb the nanoparticle formation. The choice of this solvent causes the successful formation of the MgZrO_3_ nanomaterial, which is in good accordance with the XRD data.

#### UV-DRS Analysis

The synthesized MgZrO_3_ photocatalyst’s band gap is ascertained from the diffuse reflectance spectrum depicted in Figure 1c employing Kubelka–Munk Theory. The graph between [*F*(*R*)*h*ν]^2^ and *h*ν is plotted using the relation (eq 2):
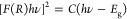
2where *h*ν represents the photon’s energy, *R* denotes the diffuse reflectance, *C* signifies the constant, and *E*_g_ represents the material’s band gap. The linear fitted region is extrapolated to achieve the band gap of the photocatalyst. The band gap of MgZrO_3_ was calculated using this theory to be ≈3.0 eV. Moreover, the considerable effect of the band gap (≈3.0 eV) on the structural attributes of MgZrO_3_ nanoparticles stands significant for the generation of electron–hole pairs. Significantly, the use of NADES solvent in the preparation of MgZrO_3_ nanoparticles exhibits the fine-tuning of the band gap, which aids the photocatalyst to perform effectively. The photocatalytic performance of the as-made MgZrO_3_ is anticipated to increase due to its separation of photogenerated carriers and the obtained band gap.

#### Morphology and Elemental Analysis

**Figure 2:**
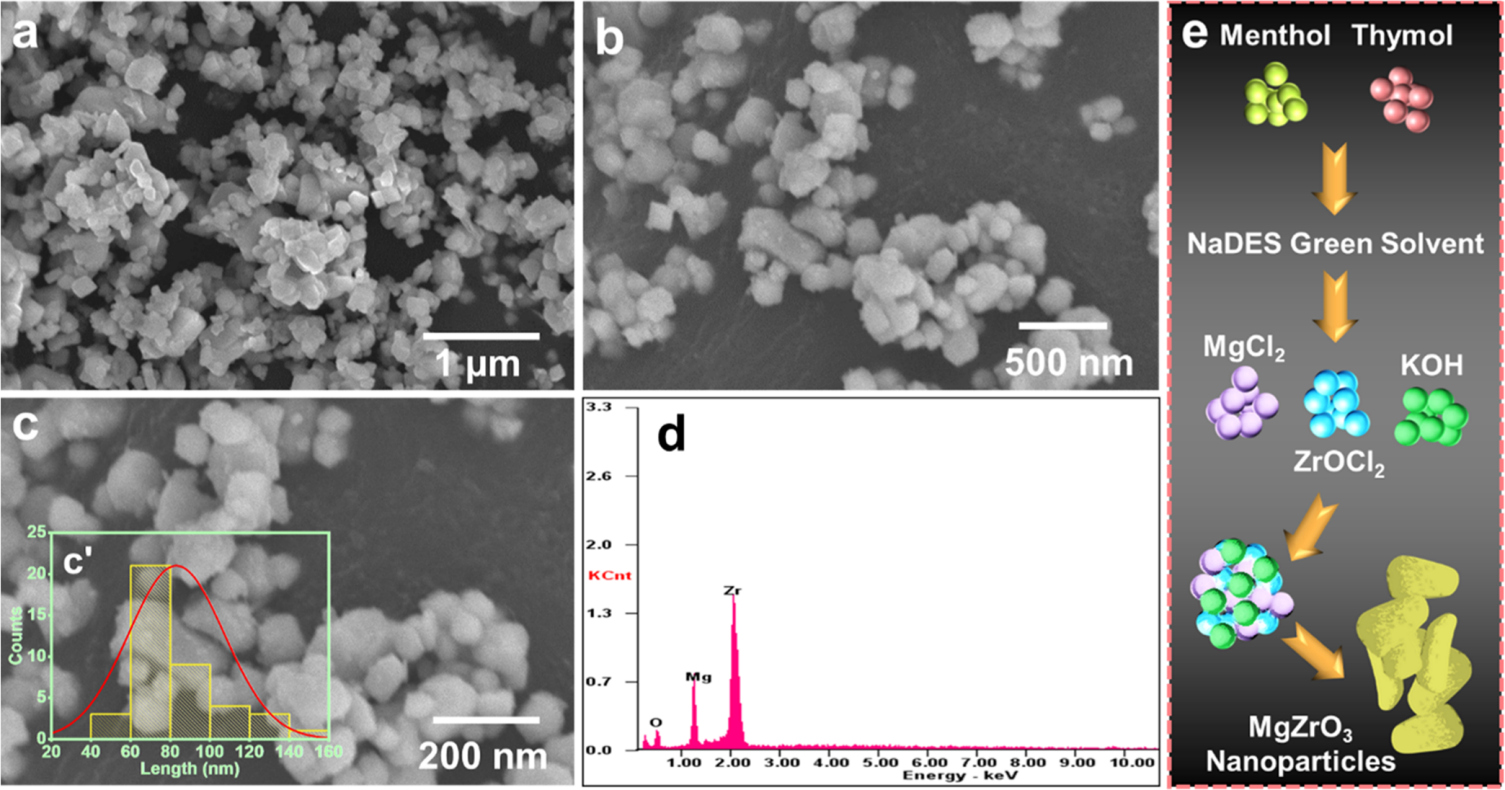
SEM images of (a, b) MgZrO_3_ nanoparticles; (c) SEM and (c’) average grain size bar diagram of MgZrO_3_ nanoparticles. (d) EDX elemental composition and (e) crystal growth pathways of MgZrO_3_ nanoparticles.

As shown in Figure 2, the structural morphology of MgZrO_3_ was investigated by employing the field emission scanning electron microscopy (FESEM) technique. It provides important details about the shape, size, and growth mechanism. Figure 2a,b displays the scanning electron microscope (SEM) imaging of MgZrO_3_ particles produced using the coprecipitation technique. The SEM images display irregular spherical MgZrO_3_ nanoparticles with clearly defined limits. These spherical nanoparticles have an increased active surface-to-volume ratio, which results in ample active sites. The high photocatalytic behavior of irregular spherical MgZrO_3_ is due to the ability of these active sites to absorb and generate electron–hole pairs. Furthermore, Figure 2c and inset c’ reveals the SEM image of MgZrO_3_ nanoparticles, and its average grain size bar diagram shows the mean value of ≈83 nm. Figure 2d displays the energy-dispersive spectroscopy (EDAX) results for MgZrO_3_. The chemical composition and dispersion of the MgZrO_3_ nanoparticles were studied by using EDAX spectroscopy. The occurrence of O (oxygen), Mg (magnesium), and Zr (zirconium) elements in the prepared sample confirms that the elemental peaks in the provided EDAX spectra are devoid of other impure peaks. Additionally, Figure 2e depicts the multistep procedure for producing MgZrO_3_ nanoparticles. Initially, menthol and thymol are combined to form the NADES green solvent. Then, magnesium chloride (MgCl_2_), zirconium oxychloride (ZrOCl_2_), and potassium hydroxide (KOH) are added to the NADES solvent. This mixture then goes through a nucleation phase, resulting in the first formation of nanoparticle seeds. Finally, the nanoparticles develop and grow into MgZrO_3_ nanoparticles.

### Photocatalytic Degradation of HCQ

#### Photocatalytic Activity of MgZrO_3_

The as-synthesized irregular nanoseed sphere-shaped MgZrO_3_ was used as an excellent photocatalyst for degrading the HCQ pollutant. The degradation efficiency of the HCQ drug was carried out in a multilamp photoreactor [Heber HML-COMPACT-SW-LW-44] fitted with four 8 W fluorescent blacklight (SANKYO DENKI F8T5/BLB) with dimensions of 28.7 cm × 1.55 cm and an output power of 1.4 W.

**Figure 3:**
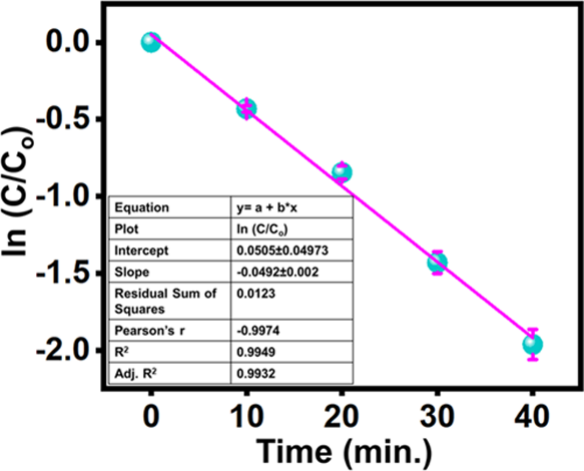
Degradation kinetics of MgZrO_3_.

The photocatalytic degradation of the HCQ experiment was conducted using 10 ppm HCQ (100 mL) solution. The as-made photocatalyst MgZrO_3_ (100 mg) was added to 100 mL of HCQ solutions. The solution was magnetically stirred in darkness for 30 min. During this period, the solution attains adsorption–desorption equilibrium. After 30 min, the photocatalytic degradation experiment of the HCQ drug molecule was conducted using a UV–visible light source. The liquid drug suspensions (4 mL) were withdrawn from the photocatalytic reactor every 10 min, respectively. The absorbance was monitored every 10 min with a UV spectrophotometer. Moreover, as the disintegration period for the nanoparticles increased, the absorption maxima of HCQ steadily decreased. The number of photodegradable radicals grows with increasing radiation time, and breakdown occurs within 30 min. After 30 min of irradiation, MgZrO_3_ reached 85% degradation. This significantly improved the derivative reduction process and triggered a redox reaction. This is favored due to its band gap, which enables an active reaction to UV–visible light followed by electron and hole photogeneration. The degradation efficiency of MgZrO_3_ is found to be 85%. Studies on the adsorption–desorption equilibrium were conducted in the dark for 30 min. This clearly shows that the absence of light causes no discernible degradation. The excellent degradation efficiency achieved by the as-made photocatalyst MgZrO_3_ was calculated using the following eq (eq 3):
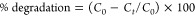
3in which *C*_0_ represents the concentration of the drug solution (initial) and *C_t_* denotes the concentration of the drug solution (at different time intervals). Furthermore, the additional optimization studies to gain a deeper understanding of the MgZrO_3_ catalytic activity are discussed below (Figure 3).

In this current study, the photocatalytic activity of the as-made NADES-assisted MgZrO_3_ was subjected to different experimental parameters such as

- Effect of catalyst dosage
- Effect of concentration of HCQ drug solutions
- Effect of pH
- Effect of scavengers
- Effect of salts
- Effect of light

#### Effect of Catalyst Dosage

**Figure 4:**
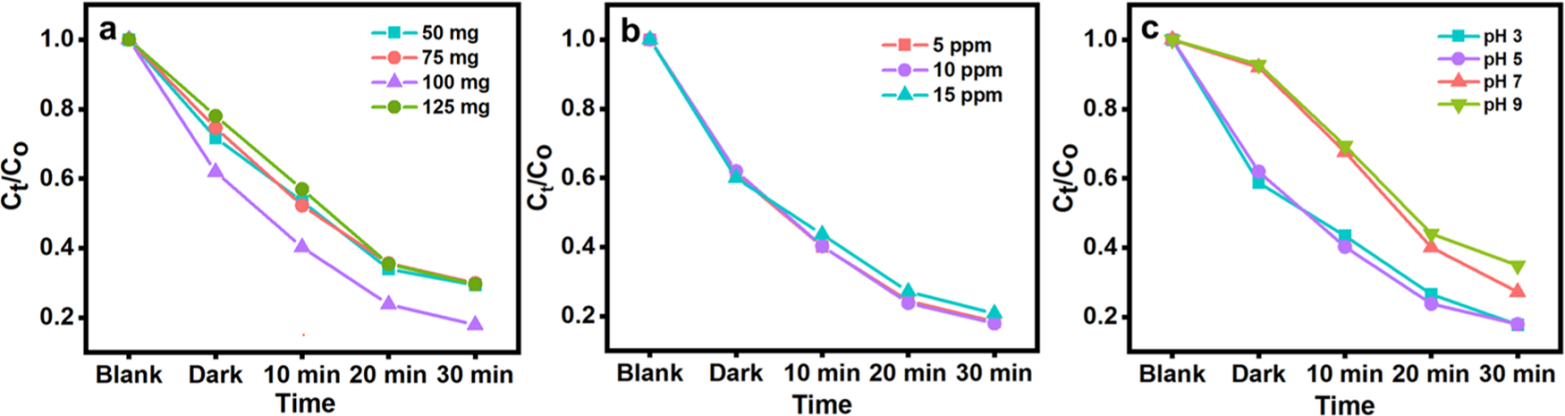
(a) Effect of MgZrO_3_ nanocatalyst dosages of 50, 75, 100, and 125 mg. (b) Effect of various HCQ concentrations of 5, 10, and 15 ppm. (c) Effect of different pH mediums of pH 3, 5, 7, and 9.

This experiment examines the impact of different MgZrO_3_ loadings toward the photocatalytic degradation of HCQ. Figure 4a shows that the HCQ drug degradation rate changes as the photocatalyst dosage varies from 50 mg/100 mL to 125 mg/100 mL in the presence of UV–visible light, maintaining the other parameter constant. From the obtained results, the absorption peak makes it evident that the photocatalytic degradation efficiency increases with an increase in the dosage of MgZrO_3_, respectively. Based on these results, the photocatalyst loading of 100 mg/100 mL (MgZrO_3_/H_2_O) is chosen to be the ideal dosage of MgZrO_3_ for HCQ degradation under UV–visible irradiation. The optimum dosage of MgZrO_3_ (100 mg/100 mL) enhances the UV–visible light absorption and increases the number of active sites that interact with the HCQ pollutant. The abundant active sites of the catalyst increase the number of OH^•^ and O^2•–^ radicals that speed up the degradation process. Further loading of the catalyst beyond the optimum point causes a reduction in the surface area that is available for the absorption of light and h^+^/e^–^ generation. After a particular point of loading, saturation occurs in the aqueous drug solution, and the MgZrO_3_ catalyst cannot be effectually suspended. As a result, the solution becomes turbid and penetration of light becomes very difficult, which further slows down the rate of degradation, respectfully. Therefore, 100 mg of the catalyst dosage works best for the HCQ degradation, reaching a maximum removal of ±85% within 30 min. As a result, this dosage was chosen as the ideal dosage of the photocatalyst.

#### Effect of Concentration

The concentration of the HCQ drug is a critical parameter, as it can impact the drug degradation efficiency. The influence of target analyte concentration was studied using 100 mg of the photocatalyst MgZrO_3_ (unchanged) with different concentrations of HCQ (5, 10, and 15 ppm), respectively. From the study, it is noted that there is a decrease in the degradation efficiency (Figure 4b) as the concentration increases from 5 to 15 ppm. This indicates that the degradation rate of the HCQ drug is inversely related to the concentration of the HCQ drug. As a result, the HCQ molecule reduces the number of accessible active sites due to competing adsorption on the MgZrO_3_ particles. This is mainly because the degradation process potentially depends upon the available active sites on the photocatalyst, which is responsible for radical production. Further, an increase in the drug concentration causes the surface of the photocatalyst (MgZrO_3_) to adsorb ample drug particles that inhibit the generation of radicals from the photocatalyst. This causes a decrease in the rate of photocatalytic degradation of the HCQ drug. Moreover, at high HCQ concentrations, the drug molecule absorbs more light irradiations, when compared to the catalysts, thereby resulting in a decreased photodegradation rate of HCQ. From this study, we conclude that the ideal concentration of HCQ drug solution is 10 ppm, respectively.

#### Effect of pH

To study the influence of pH on the photocatalytic degradation of HCQ, experiments were carried out with the most efficient catalyst, MgZrO_3_, by altering the initial pH of the solution (5) to a more acidic solution (3), to a neutral solution (7), and to a more basic solution (9), at a concentration of 10 ppm irradiated by the UV–visible lamp. Figure 4c depicts the photocatalytic degradation of HCQ at different pH. The observation shows that at the current pH 5 of the drug solution, the as-prepared MgZrO_3_ undergoes protonation that helps in the thermodynamically achievable degradation process. At pH 5, MgZrO_3_ undergoes enhanced charge separation owing to its decelerated electron–hole pair recombination process. Hence at pH 5, there is increased HCQ degradation. Further changing the pH from 5 to 3 by adding HCl acid to the original drug solution slightly decreases the degradation efficiency from the pH 5 degradation profile. This is because the photocatalyst and the HCQ drug moiety become positively charged and repel each other. Therefore, low degradation efficiency is observed for pH 3. As the pH increases to 7 and 9, there can be a development of resistance between the catalyst surface and the HCQ molecule. At this point, the HCQ drug moiety undergoes deprotonation and the surface of MgZrO_3_ is negatively charged, leading to the lowering of degradation efficiency of the HCQ drug. This efficiency decrease in the photodegradation of the HCQ drug is explained by the reduced accessibility of the HCQ drug (cationic form) and enhanced OH^–^ ion concentration, lowering the Coulombic force of attraction between the MgZrO_3_ surface and the drug species. Hence, the pH investigation of the drug solution concludes that pH 5 is the optimum pH for the photocatalytic degradation of HCQ.

#### Effect of Scavengers

**Figure 5:**
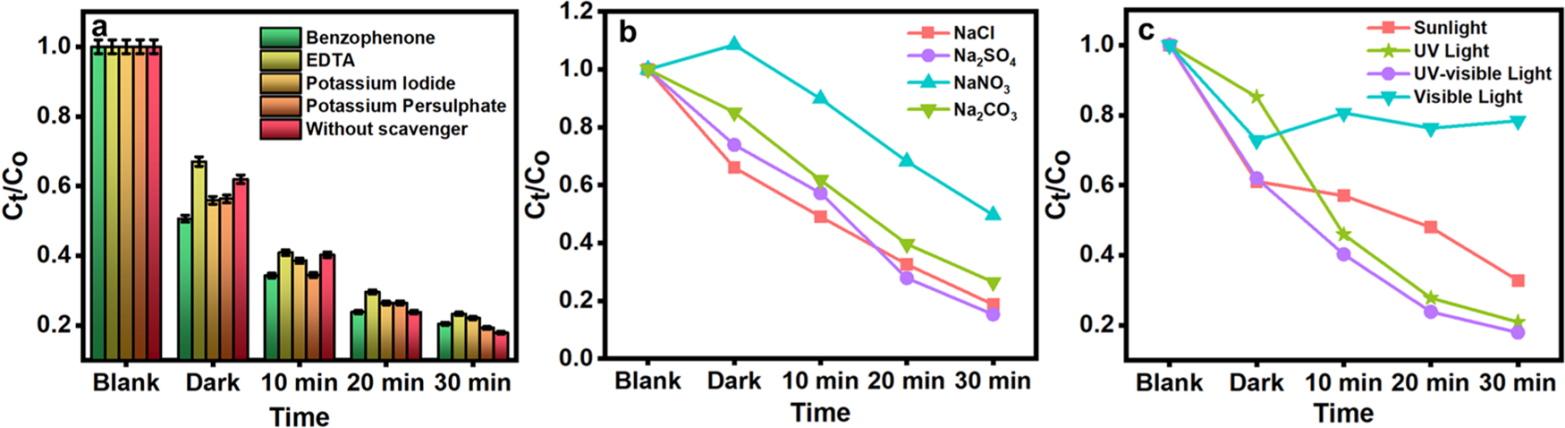
(a) Effect of scavengers: benzophenone, ethylenediaminetetraacetic acid (EDTA), potassium iodide, potassium persulfate, and without scavenger. (b) Effect of salts: NaCl, Na_2_SO_4_, NaNO_3_, and Na_2_CO_3_. (c) Effect of light sources: sunlight, UV light, UV–visible light, and visible light.

The significant approach for explicating the photocatalysis of drug pollutants by employing scavengers to trap holes and free electrons is considered advantageous. The contribution of each trapping agent toward the photocatalytic activity of MgZrO_3_ nanoparticles for the degradation of the HCQ drug is given in Figure 5a. The scavengers selected for the analysis are EDTA, benzophenone, potassium iodide, and potassium persulfate. The results reveal that the photocatalytic degradation of the HCQ drug is very slightly affected by the scavenger (benzophenone, EDTA, and potassium persulfate) and remains unaffected by the potassium iodide. These outcomes infer that the degradation efficiency is slightly hindered by adding benzophenone signifying the influence of ^•^O_2_^–^ in the HCQ degradation process. The addition of EDTA causes the degradation efficiency of the HCQ drug to decrease marginally, owing to the small effect of h^+^ in the reaction process. Further, when potassium persulfate was added, the degradation efficiency of the HCQ was found to reduce accrediting to the key influence of ^•^OH in the HCQ photodegradation. The ^•^OH active species are accountable for the formation of a large number of holes. The occurrence of air oxygen can bestow to photocatalytic oxidation. Oxygen is the significant electron acceptor that restrains the recombination of electron–hole pairs, stimulating the generation of OH radicals. Thus, the resultant outcome of the scavenging studies affirmed the predominance of OHs in HCQ drug degradation.

#### Effect of Salts

The salinity effect influences drug degradation efficiency. The salting out effect was studied by adding 0.5 g of sodium chloride (NaCl), sodium sulfate (Na_2_SO_4_), sodium carbonate (Na_2_ CO_3_), and sodium nitrate (NaNO_3_) aqueous solutions. Figure 5b reveals a significant increase in the drug removal efficiency of the MgZrO_3_ nanoparticles. The investigation reveals that the Na_2_SO_4_ salt aids the adsorption of HCQ on the surface of the photocatalyst. The addition of Na_2_SO_4_ salt influences the degradation process, as it plays two major significant roles.

(a) According to the adsorption phenomenon, the distribution of the drug molecule changes over the surface of the photocatalyst (MgZrO_3_) as the addition of Na_2_SO_4_ salt alters the charges on the MgZrO_3_ surface.
(b) The adsorbed anions from the Na_2_SO_4_ salt undergo a reaction with the holes and hydroxide radicals producing reactive species in the drug solution.

Further, the solubility of the drug in an aqueous solution is restricted as the amount of Na_2_SO_4_ molecules upsurges. The decreased drug solubility is due to the salting out effect of the Na_2_SO_4_ salt, causing increased drug molecules to diffuse to the MgZrO_3_ surface. This increases the adsorption efficiency. The obtained outcome from the salinity study reveals the increased HCQ drug elimination in the presence of Na_2_SO_4_ salt.

#### Effect of Light Sources

The intensity and wavelength of the light sources impact the photocatalytic activity of the as-prepared MgZrO_3_ toward the degradation of the HCQ drug. Therefore, the influence of different light irradiations toward the photocatalytic degradation of the HCQ drug was examined by employing distinctive light sources such as natural sunlight, UV light, and visible light. Figure 5c reveals the degradation graph of the 10 ppm HCQ drug under the solar, UV, and visible radiations in the presence of 100 mg of catalyst. Among the UV light, visible light, and UV–visible light sources, it is noted that the UV–visible light sources (used in the above-mentioned parameters) aided in the rapid degradation of the HCQ drug with an enhanced degradation efficiency of ±85%. This is due to the penetrating power of the light sources. The penetrating power is meager for the shorter wavelength light sources with high energies. Compared to the UV and visible light sources, UV–visible light (longer wavelength range) was a more efficient source for photocatalytic degradation of the HCQ drug. Furthermore, the energy of the UV–visible irradiation is higher than the band gap of the as-synthesized photocatalyst. The band gap of MgZrO_3_ is 3.0 eV and remarkable optical properties such as its high optical transparency in the visible range, which makes it an efficient degradation catalyst under UV–vis light. Therefore, the hindrance regarding electron–hole recombination is mostly circumvented with the UV–visible light source. Nevertheless, the overall sunlight radiation consists of only 5% of the ideal UV energy required for the excitation of the electrons. Thus, photocatalytic degradation of HCQ drug is found to be less, and it takes a longer time in solar radiation. Hence, in the presence of catalysts, the degradation of HCQ was greater and faster with the UV–visible light.

#### Plausible Photocatalytic Degradation Mechanism

**Scheme 2:**
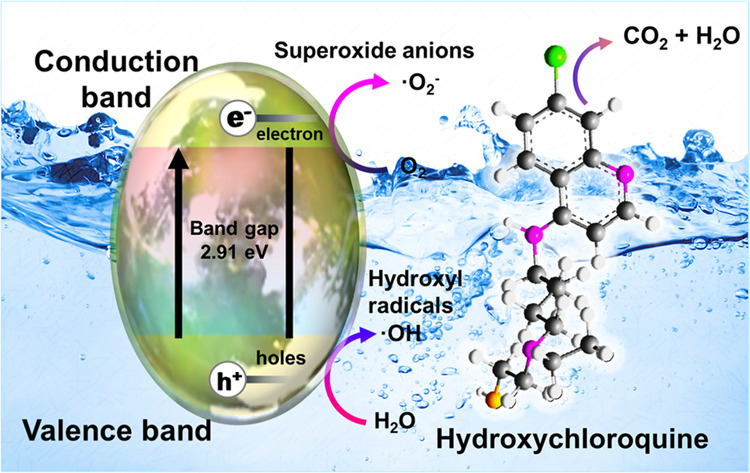
Plausible Photocatalytic Degradation Mechanism

Typically, the reaction mechanism encompassing the photocatalytic degradation of the HCQ molecule is explained as follows. The as-prepared MgZrO_3_ (band gap of ≈3.0 eV) photocatalyst is excited in the presence of UV–visible light irradiation, producing electron–hole pairs on the surface of the as-prepared nanomaterial. Elaborating on the process, due to UV–visible light excitation, the electrons (e^–^) hop and reach the conduction band (CB) from the valence band (VB). This step results in the generation of unoccupied h^+^ holes in the valence band. This process is explained in eq 5. The electrons produced from the photocatalyst can reduce oxygen molecules to reactive ^•^O_2_^–^ radicals. According to eqs 7 and 8, these free radicals (^•^O_2_^–^) react with the proton to produce ^•^HO_2_. As per the equation, the valence band with h^+^ holes reacts with the photocatalyst (MgZrO_3_) in water to produce OH^•^ radicals. Thus, in this catalytic reaction, complete reactive oxygen species (ROS) were generated in the presence of UV–visible light irradiation. This imparts the possible photocatalytic degradation of the HCQ molecule.^33,34^ The plausible degradation mechanism of the HCQ drug by the as-prepared photocatalyst is demonstrated in Scheme 2 and eq 4–9.
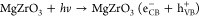
4
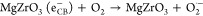
5
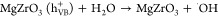
6
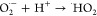
7
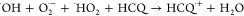
8
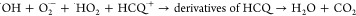
9

## Conclusions

Herein, our goal is to curate a new synthetic procedure for reproducible synthesis of MgZrO_3_ nanoparticles using an environmentally friendly thymol–menthol NADES system to provide a highly efficient UV–visible light-driven photocatalyst for effective photocatalytic degradation of the HCQ drug. The structural characterizations reveal the supreme features of the as-prepared material. The XRD and FTIR results indicated that the as-prepared MgZrO_3_ nanoparticles were highly pure and crystalline with corresponding functional stretching vibrations. Based on the SEM analysis, the distinctive nanoseed architecture enabled a rapid charge transfer process that enhanced the photocatalytic activity of the as-prepared MgZrO_3_. The relevant reaction parameters, including catalyst dosage, HCQ concentration, pH, scavengers, and salinity studies, are optimized experimentally. The obtained outcomes indicated that ±85% of HCQ (10 ppm) was degraded using a MgZrO_3_ photocatalyst (100 mg) at pH 5 within 30 min of the reaction time under UV–visible light irradiation. The harmful HCQ residues interact with the ^•^OH radicals formed during the photocatalytic process on the surface of the as-synthesized MgZrO_3_ nanoparticle resulting in pseudo-first-order kinetics of the HCQ degradation. respectively. Hence, the inclusion of UV–visible irradiation, as the source of activation, makes the process relevant for the cost-effective and environment-friendly toward removal of HCQ residues from water. The main merits of this study include the formation of MgZrO_3_ nanoparticles at possibly low calcination temperature at about 600 °C using the innovative synthesis route. To the best of the authors’ knowledge, this is the first report on the photocatalytic degradation of HCQ using only MgZrO_3_ nanoparticles as a photocatalyst. Significantly, the present study displayed an effective drug removal approach that is anticipated to enhance adherence to stringent policy agreements and enforcement, thereby addressing health issues related to the dumping of hazardous pharmaceutical waste in aquatic environments.

## Supporting Information Available

Comparison table of degradation of HCQ, and references (PDF)
