# Supplementary material for: Sustainable Natural Deep Eutectic Solvent-Mediated Synthesis of Magnesium Zirconate Nanoparticles: A Photocatalyst for the Degradation of Anti-Viral Drug
Source: Inorg Chem. 2024 Oct 11;63(43):20705–13. doi: 10.1021/acs.inorgchem.4c03383 (PMC11523255; doi:10.1021/acs.inorgchem.4c03383)
Supplement: Supplementary file 1 — ic4c03383_si_001.pdf [file ic4c03383_si_001.pdf]

Supporting Information

Sustainable Natural Deep Eutectic Solvent-Mediated Synthesis of Magnesium Zirconate Nanoparticles: A Photocatalyst for the Degradation of Anti-Viral Drug

Balasubramanian Sriram[a], *Abhikha Sherlin V*[b], *Sea–Fue Wang*[a,*], *Jackulinflora P*[b], *Mary George*[b]

[a]*Department of Materials and Mineral Resources Engineering, National Taipei University of Technology, No. 1, Section 3, Chung-Hsiao East Road, Taipei 106, Taiwan.*

[b]*Department of Chemistry, Stella Maris College, Affiliated to the University of Madras, Chennai, Tamil Nadu 600086, India.*

Corresponding author:

Dr. Sea–Fue Wang, E-mail: sfwang@ntut.edu.tw

No. of pages: 2

No. of Table: 1

Table of content entry

Contents **Page No.**

Table S1. Comparison table of degradation of HCQ……………….. S2
References……………………………………………………… S2

Table S1. Comparison table of degradation of HCQ.

**Catalyst**	**Method**	**Degradation efficiency (%)**	**Time (Min.)**	**Ref.**
MoS_2/CNTs	Photocatalytic	70 %	120	S1
Ti_3GeC_2 with peroxydisulfate	Photocatalytic	60.42 %	80	S2
**$MgZrO_3$**	**Photocatalytic**	**85%**	**30**	**This Work**

Reference

S1. Dastborhan, M., Khataee, A., Arefi-Oskoui, S. and Yoon, Y., 2022. Synthesis of flower-like MoS2/CNTs nanocomposite as an efficient catalyst for the sonocatalytic degradation of hydroxychloroquine. Ultrasonics Sonochemistry, 87, p.106058.

S2. Ansarian, Z., Khataee, A., Arefi-Oskoui, S., Orooji, Y. and Lin, H., 2022. Ultrasound-assisted catalytic activation of peroxydisulfate on Ti3GeC2 MAX phase for efficient removal of hazardous pollutants. Materials Today Chemistry, 24, p.100818.
